# Supplementary material for: Degradation Profiling of Nardosinone at High Temperature and in Simulated Gastric and Intestinal Fluids
Source: Molecules. 2023 Jul 13;28(14):5382. doi: 10.3390/molecules28145382 (PMC10385092; doi:10.3390/molecules28145382)
Supplement: Supplementary file 1 [file molecules-28-05382-s001.zip › molecules-2455563-supplementary.pdf]

Supplementary Material

Degradation Profiling of Nardosinone at High Temperature, and in Simulated Gastric and Intestinal Fluids

Bian-Xia Xue[a,†], Tian-Tian Yang[a,†], Ru-Shang He[a,†], Wen-Ke Gao[a], Jia-Xin Lai[a], Si-Xia Liu[a], Cong-Yan Duan[a], Shao-Xia Wang[a], Hui-Juan Yu[a], Wen-Zhi Yang[a], Li-Hua Zhang[a,*], Qi-Long Wang[a,*], Hong-Hua Wu[a,b,*]

[a] *National Key Laboratory of Chinese Medicine Modernization, State Key Laboratory of Component-Based Chinese Medicine, Tianjin University of Traditional Chinese Medicine, 10 Poyanghu Road, West Area, Tuanbo New Town, Jinghai District, Tianjin 301617, China*

[b] *Tianjin Key Laboratory of Therapeutic Substance of Traditional Chinese Medicine, Tianjin University of Traditional Chinese Medicine, 10 Poyanghu Road, West Area, Tuanbo New Town, Jinghai District, Tianjin 301617, China*

---

* **Corresponding authors:**

Li-Hua Zhang, Institute of Traditional Chinese Medicine, Tianjin University of Traditional Chinese Medicine, 10 Poyanghu Road, West Area, Tuanbo New Town, Jinghai District, Tianjin, 301617, China

Tel./fax: +86-022-59791830

E-mail address: zhanglihua200061@163.com (L.-H. Zhang).

Qi-Long Wang, Institute of Traditional Chinese Medicine, Tianjin University of Traditional Chinese Medicine, 10 Poyanghu Road, West Area, Tuanbo New Town, Jinghai District, Tianjin, 301617, China

E-mail address: wangqilong_00@tjutcm.edu.cn (Q.-L. Wang).

ORCID: https://orcid.org/0000-0002-4375-991X

Hong-Hua Wu, Institute of Traditional Chinese Medicine, Tianjin University of Traditional Chinese Medicine, 10 Poyanghu Road, West Area, Tuanbo New Town, Jinghai District, Tianjin, 301617, China

Tel./fax: +86-022-59791833

E-mail address: wuhonghua2011@tjutcm.edu.cn (H.-H. Wu).

ORCID: https://orcid.org/0000-0002-3089-4282.

† **Co-first authors:**

B. X. Xue, T.-T. Yang and R.-S. He contributed equally to this work and should be considered co-first authors

Table of Contents		Page
**Figure S1**	Structures (**a**) and optimized conformers (**b**) for the ECD calculation of 2–deoxokanshone M	**2**
**Figure S2**	Structures (**a**) and optimized conformers (**b**) for the ECD calculation of 2–deoxokanshone L	**2**
**Figure S3**	Representative UPLC chromatograms of 2–deoxokanshone M incubated in HT and SGF–A	**3**
**Figure S4**	The stability of 2–deoxokanshone M in boiling water for 2 h. (**a**) Stacked UPLC chromatograms of 2–deoxokanshone M in boiling water for 0, 30, 60, and 120 min (**b**); the line graph of the relative content change of nardosinone and 2–deoxokanshone M in boiling water	**3**
**Figure S5**	^{1}H NMR (600 MHz, CD_3OD) spectrum of 2–deoxokanshone M	**4**
**Figure S6**	^{13}C NMR (150 MHz, CD_3OD) spectrum of 2–deoxokanshone M	**4**
**Figure S7**	^{1}H NMR (600 MHz, CD_3OD) spectrum of the reaction mixture of 2–deoxokanshone M in 50% MeOH at 80°C for 48 h	**5**
**Figure S8**	Representative UPLC chromatograms of nardosinone incubated in SGF (**a**), SGF–A (**b**) and SGF–B (**c**)	**6**
**Figure S9**	The degradation rate and dynamic variations of nardosinone and degradation products under three different conditions for 108 h. (**a**) Degradation percent of nardosinone in three conditions; time-concentration curves of nardosinone and its main degradation products in SGF (**b**), SGF–A (**c**) and SGF–B (**d**)	**6**
**Figure S10**	Mass spectrum ion fragment of desoxo-narchinol A	**7**
**Figure S11**	Mass spectrum ion fragment of 2–deoxokanshone L	**7**
**Figure S12**	Mass spectrum ion fragment of nardosinonediol	**8**
**Figure S13**	Mass spectrum ion fragment of isonardosinone	**8**
**Figure S14**	Mass spectrum ion fragment of 2–deoxokanshone M	**9**
**Figure S15**	Mass spectrometry fragmentation pathway of desoxo-narchinol A (positive ion mode)	**10**
**Figure S16**	Mass spectrometry fragmentation pathway of 2–deoxokanshone L (positive ion mode)	**11**
**Figure S17**	Mass spectrometry fragmentation pathway of nardosinonediol (positive ion mode)	**12**
**Figure S18**	Mass spectrometry fragmentation pathway of isonardosinone (positive ion mode)	**13**
**Figure S19**	Mass spectrometry fragmentation pathway of 2–deoxokanshone M (positive ion mode)	**14**
**Figure S20**	The separation process diagram of nardosinone	**15**
**Table S1**	Characterization of nardosinone and its degradation products in three different conditions by using UHPLC-DAD/ESI–Q–TOF MS	**16**
**Table S2**	The detailed parameters of the NMR measurements	**19**

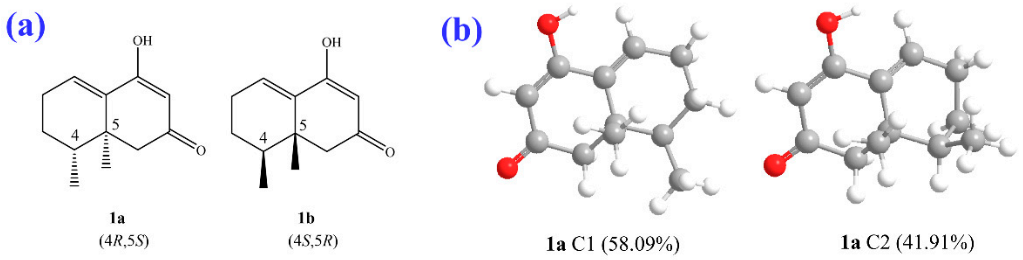

Figure S1 Structures (**a**) and optimized conformers (**b**) for the ECD calculation of 2–deoxokanshone M

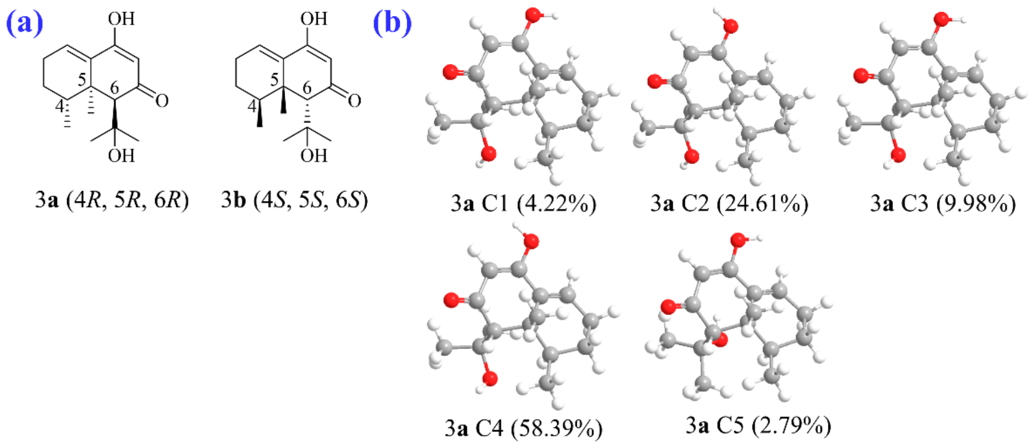

Figure S2 Structures (**a**) and optimized conformers (**b**) for the ECD calculation of 2–deoxokanshone L

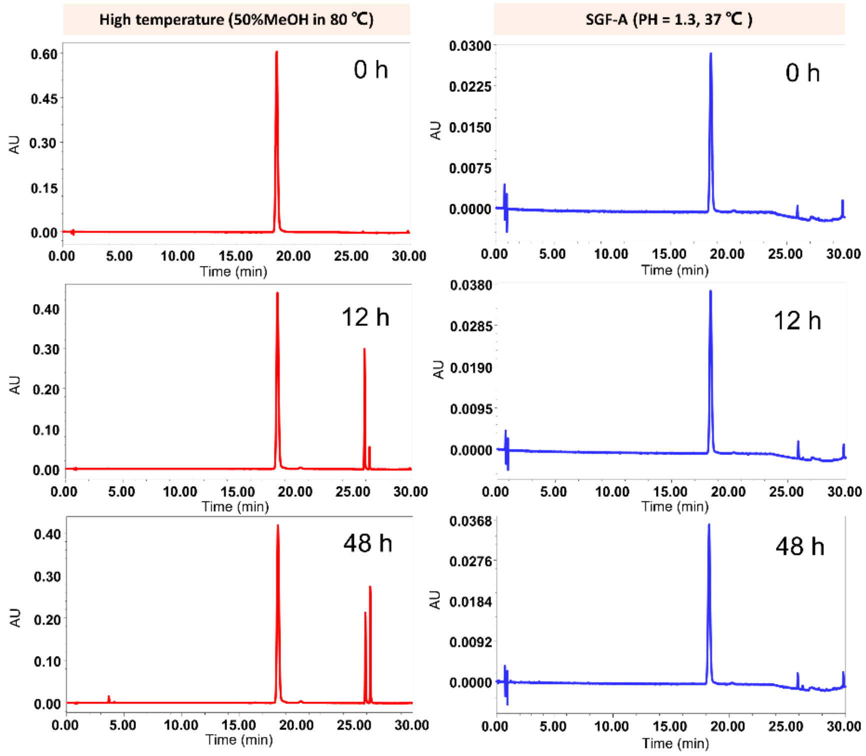

Figure S3 Representative UPLC chromatograms of 2–deoxokanshone M incubated in HT and SGF–A

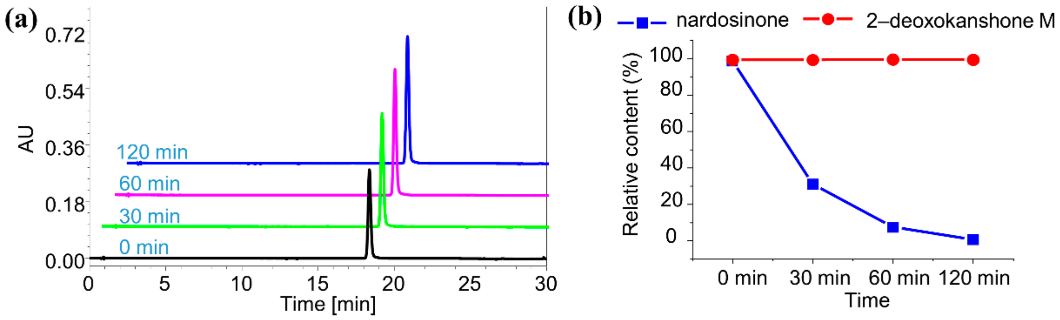

Figure S4 The stability of 2–deoxokanshone M in boiling water for 2 h. (**a**) Stacked UPLC chromatograms of 2–deoxokanshone M in boiling water for 0, 30, 60, and 120 min (**b**); the line graph of the relative content change of nardosinone and 2–deoxokanshone M in boiling water

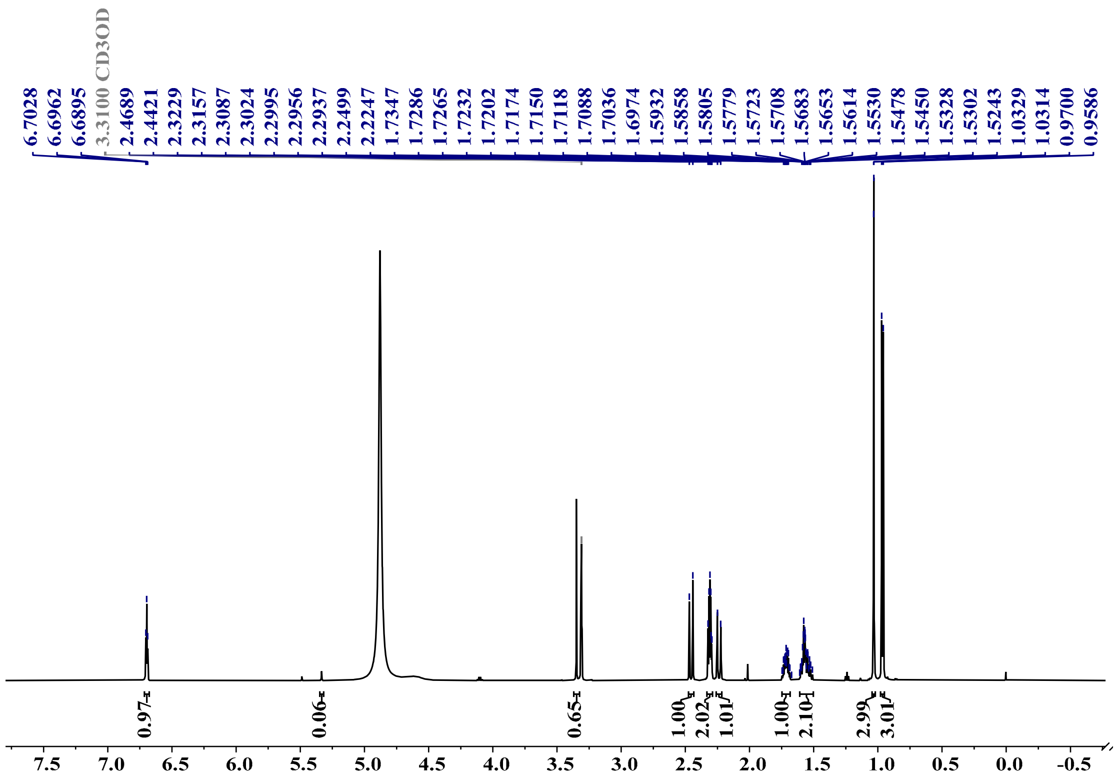

Figure S5 ^{1}H NMR (600 MHz, CD_3OD) spectrum of 2–deoxokanshone M

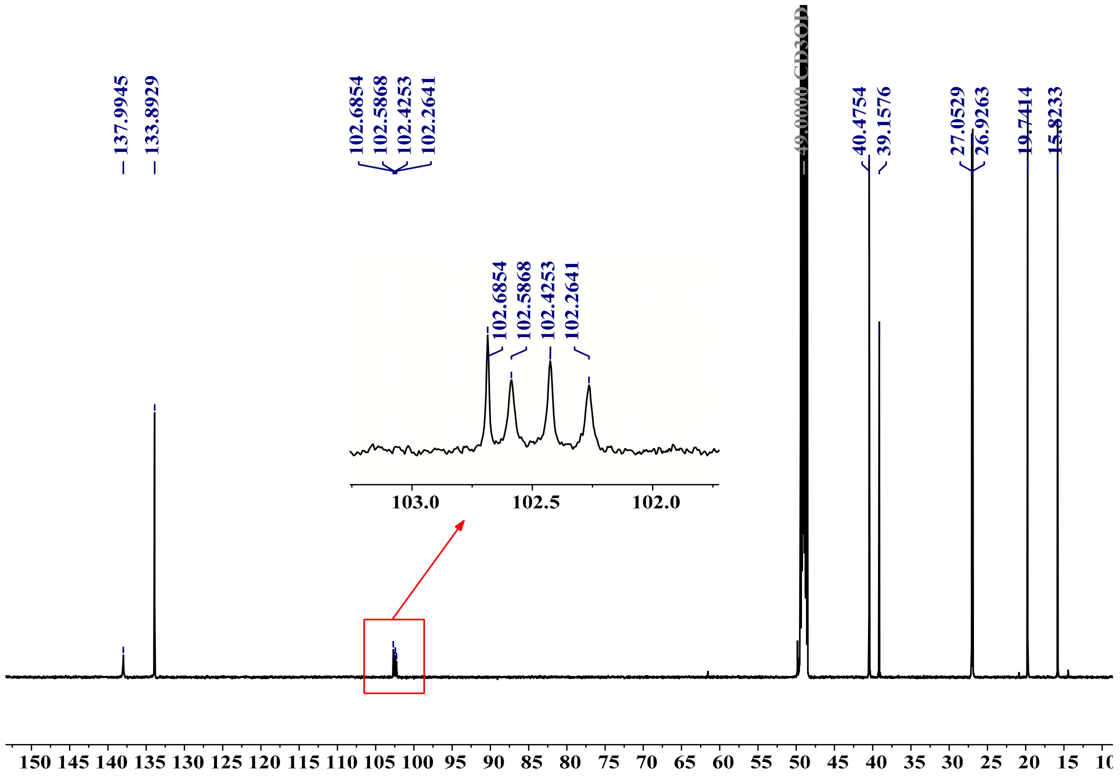

Figure S6 ^{13}C NMR (150 MHz, CD_3OD) spectrum of 2–deoxokanshone M

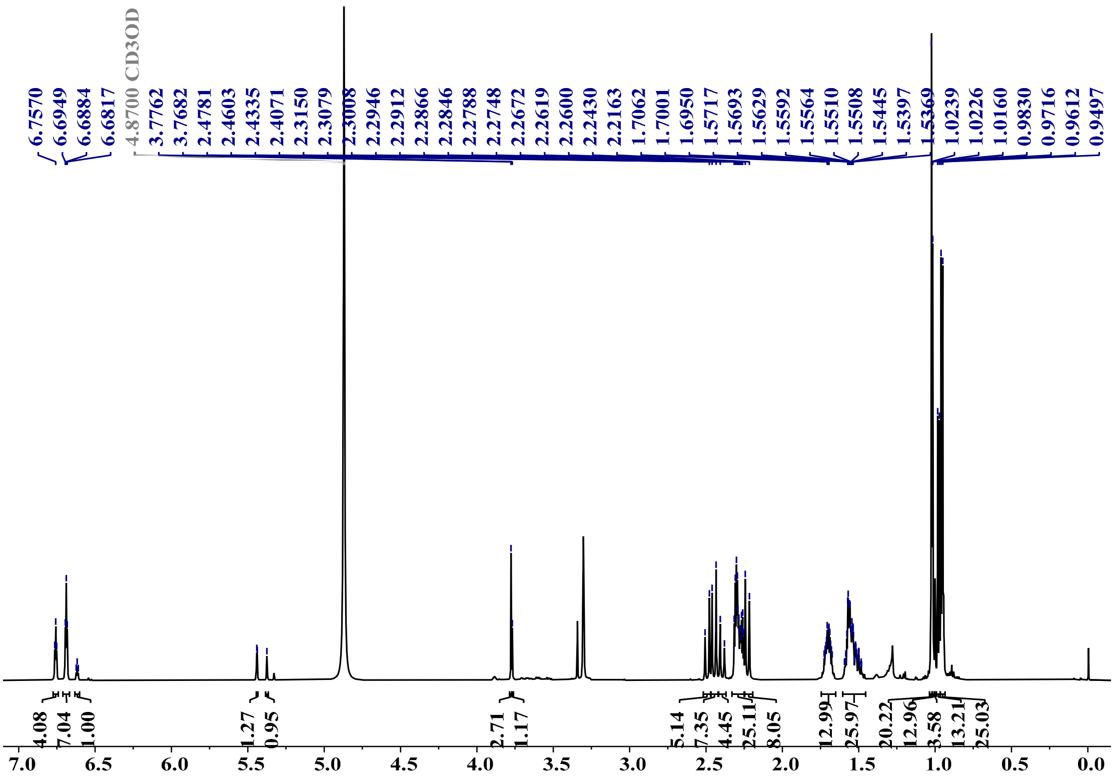

Figure S7 ^{1}H NMR (600 MHz, CD_3OD) spectrum of the reaction mixture of 2–deoxokanshone M in 50% MeOH at 80°C for 48 h

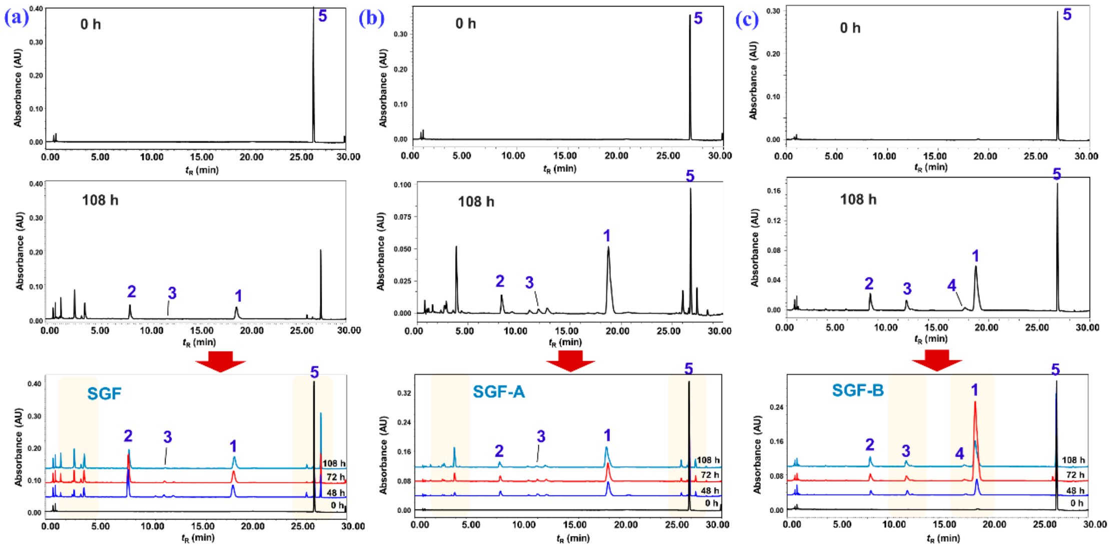

Figure S8 Representative UPLC chromatograms of nardosinone incubated in SGF (**a**), SGF–A (**b**) and SGF–B (**c**)

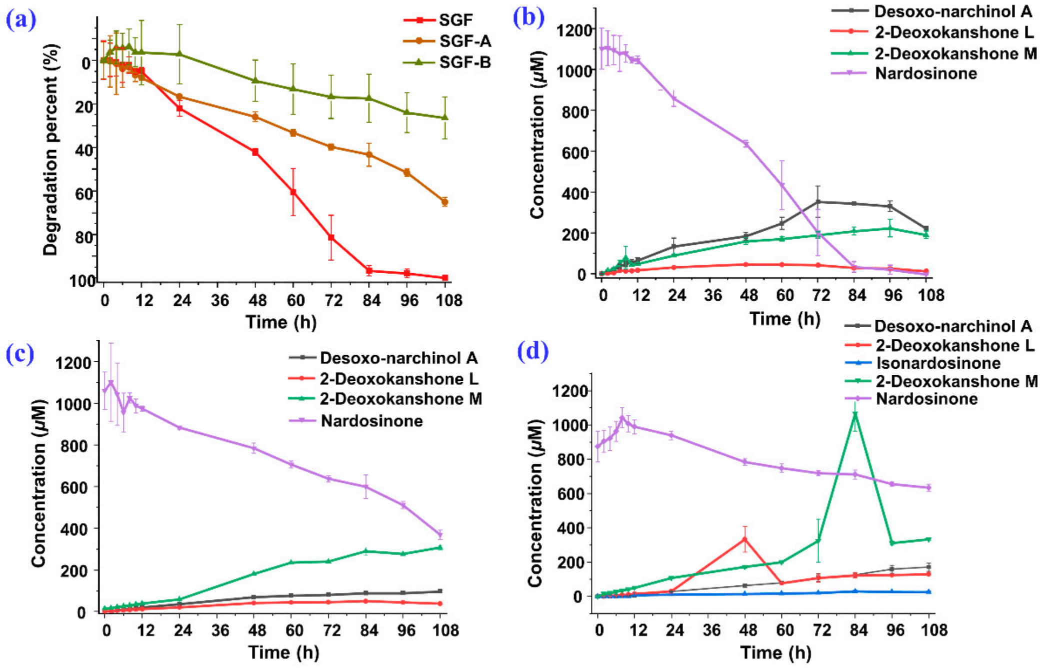

Figure S9 The degradation rate and dynamic variations of nardosinone and degradation products under three different conditions for 108 h. (**a**) Degradation percent of nardosinone in three conditions; time-concentration curves of nardosinone and its main degradation products in SGF (**b**), SGF–A (**c**), and SGF–B (**d**)

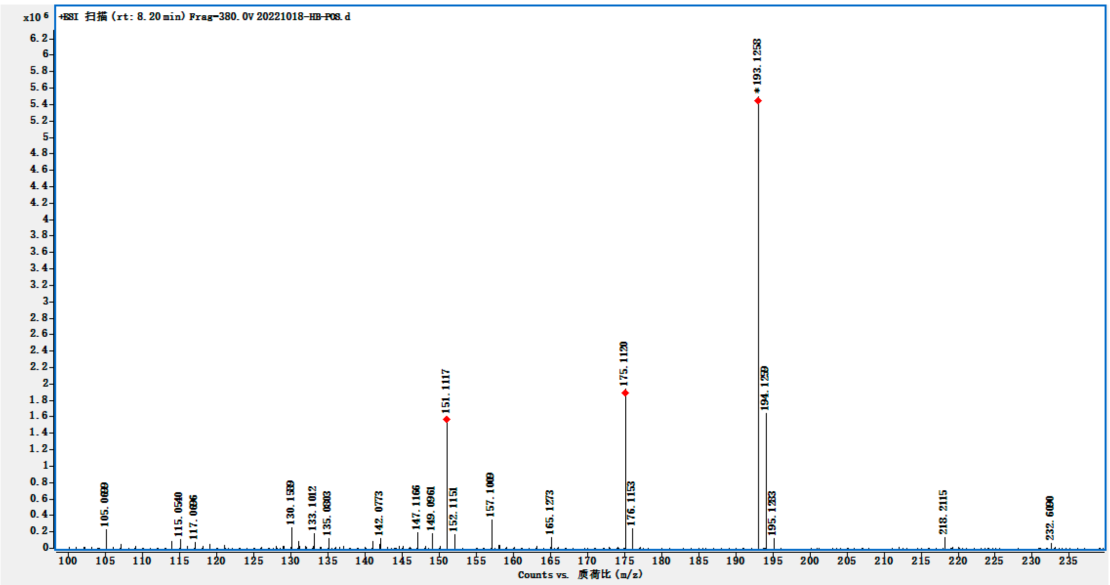

Figure S10 Mass spectrum ion fragment of desoxo-narchinol A

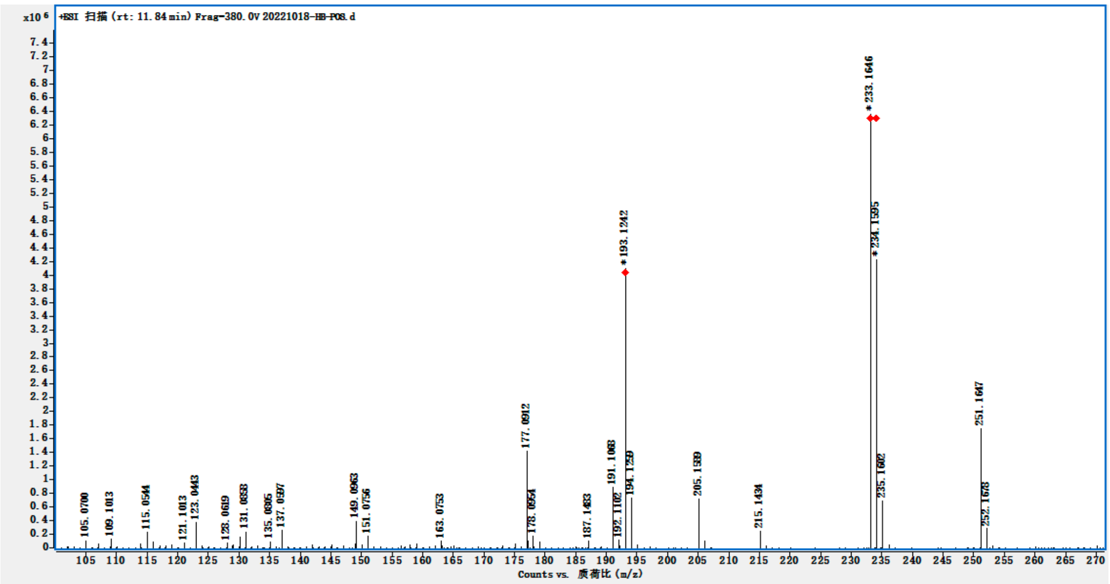

Figure S11 Mass spectrum ion fragment of 2–deoxokanshone L

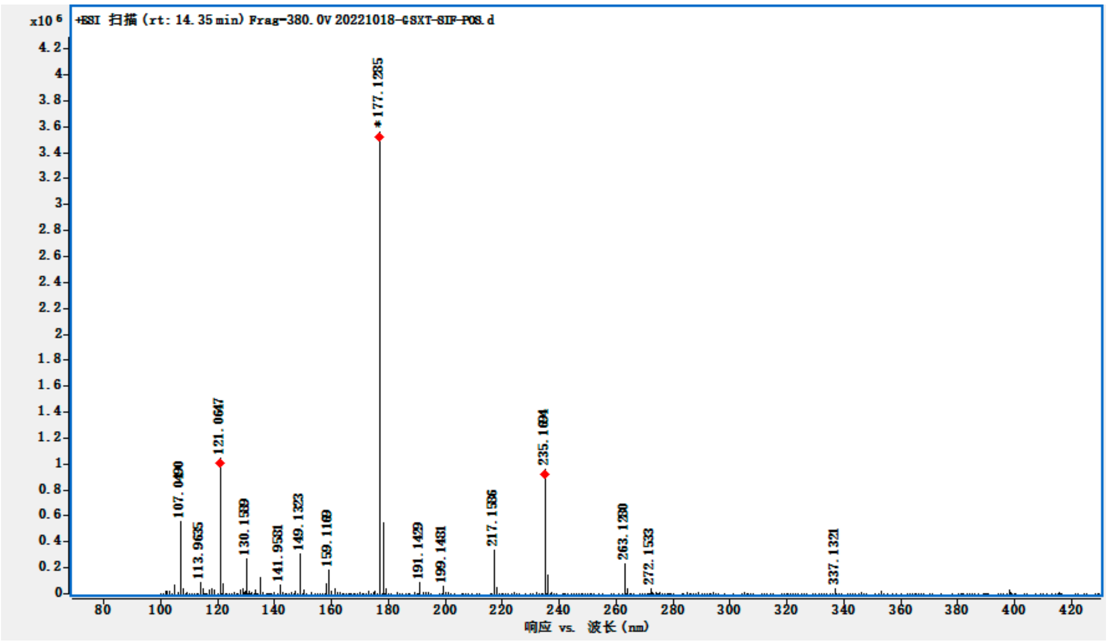

Figure S12 Mass spectrum ion fragment of nardosinonediol

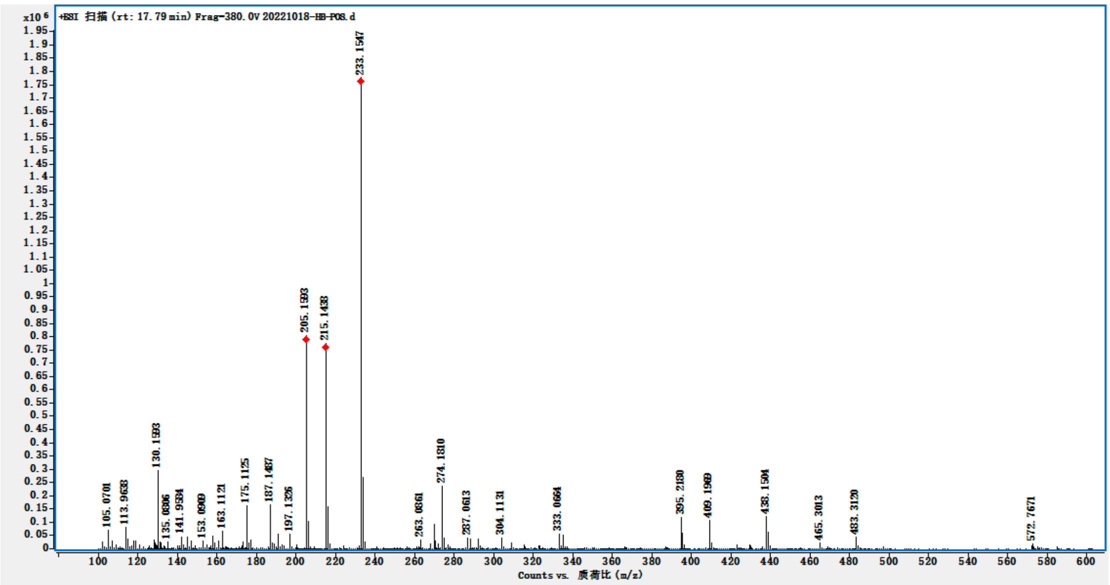

Figure S13 Mass spectrum ion fragment of isonardosinone

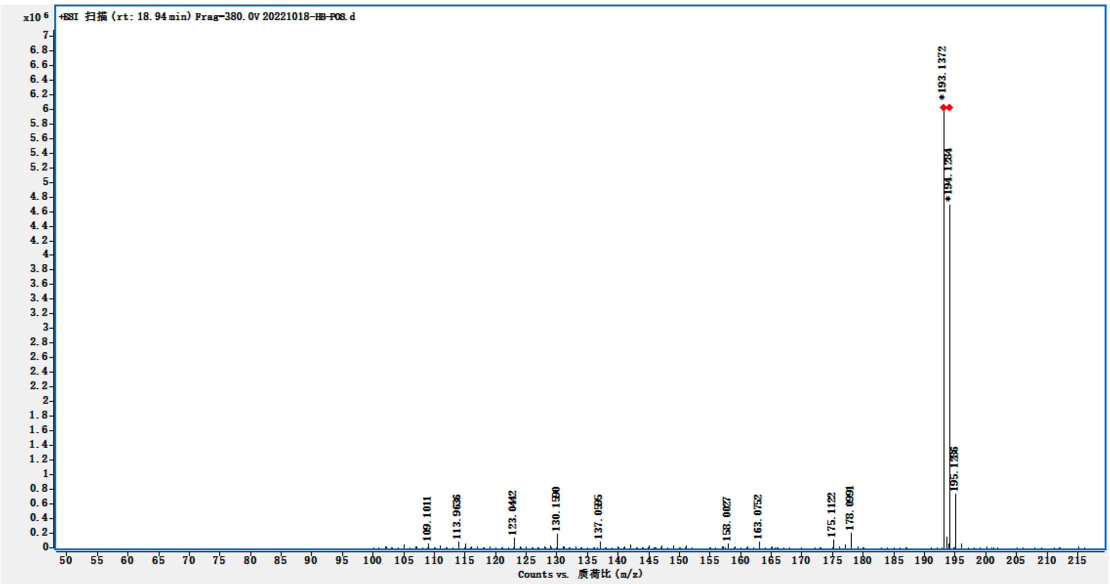

Figure S14 Mass spectrum ion fragment of 2–deoxokanshone M

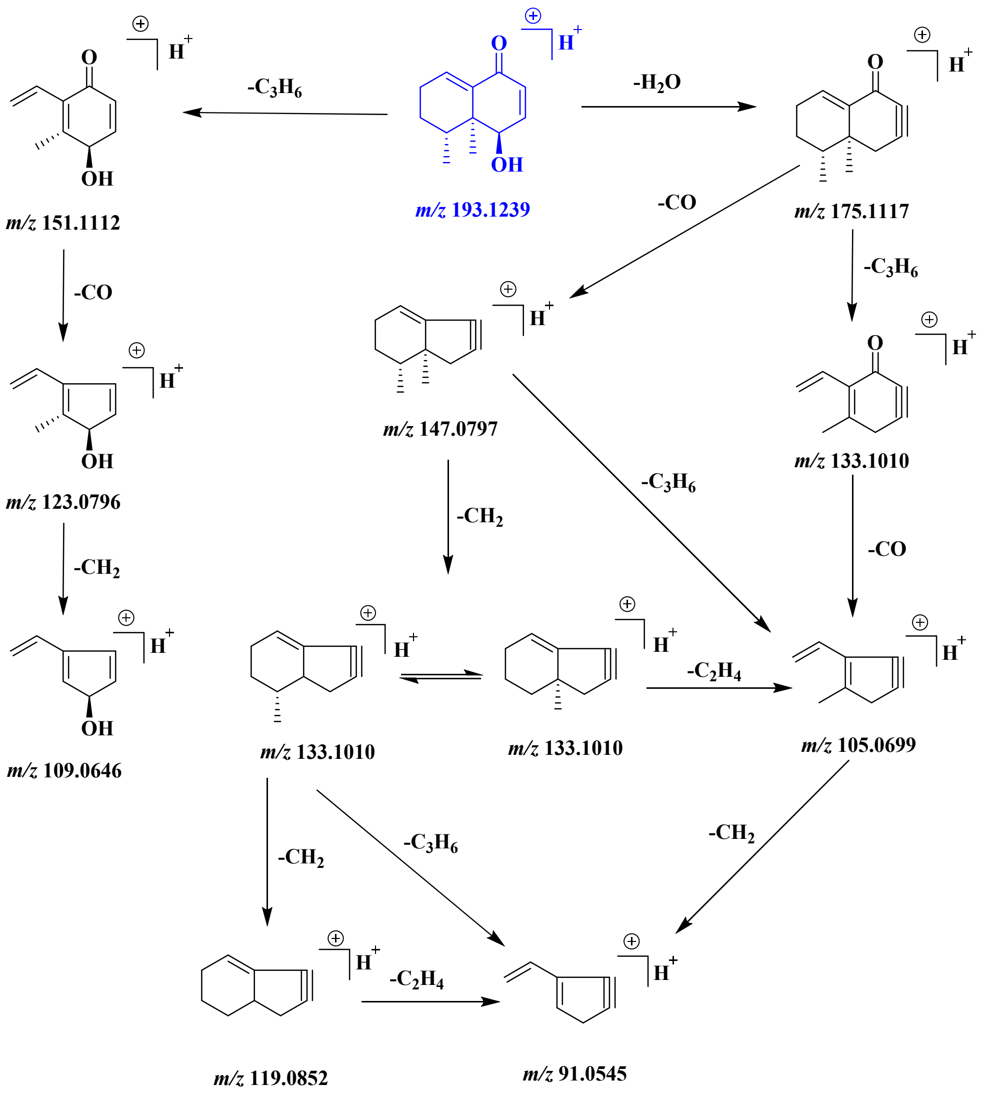

Figure S15 Mass spectrometry fragmentation pathway of desoxo-narchinol A (positive ion mode)

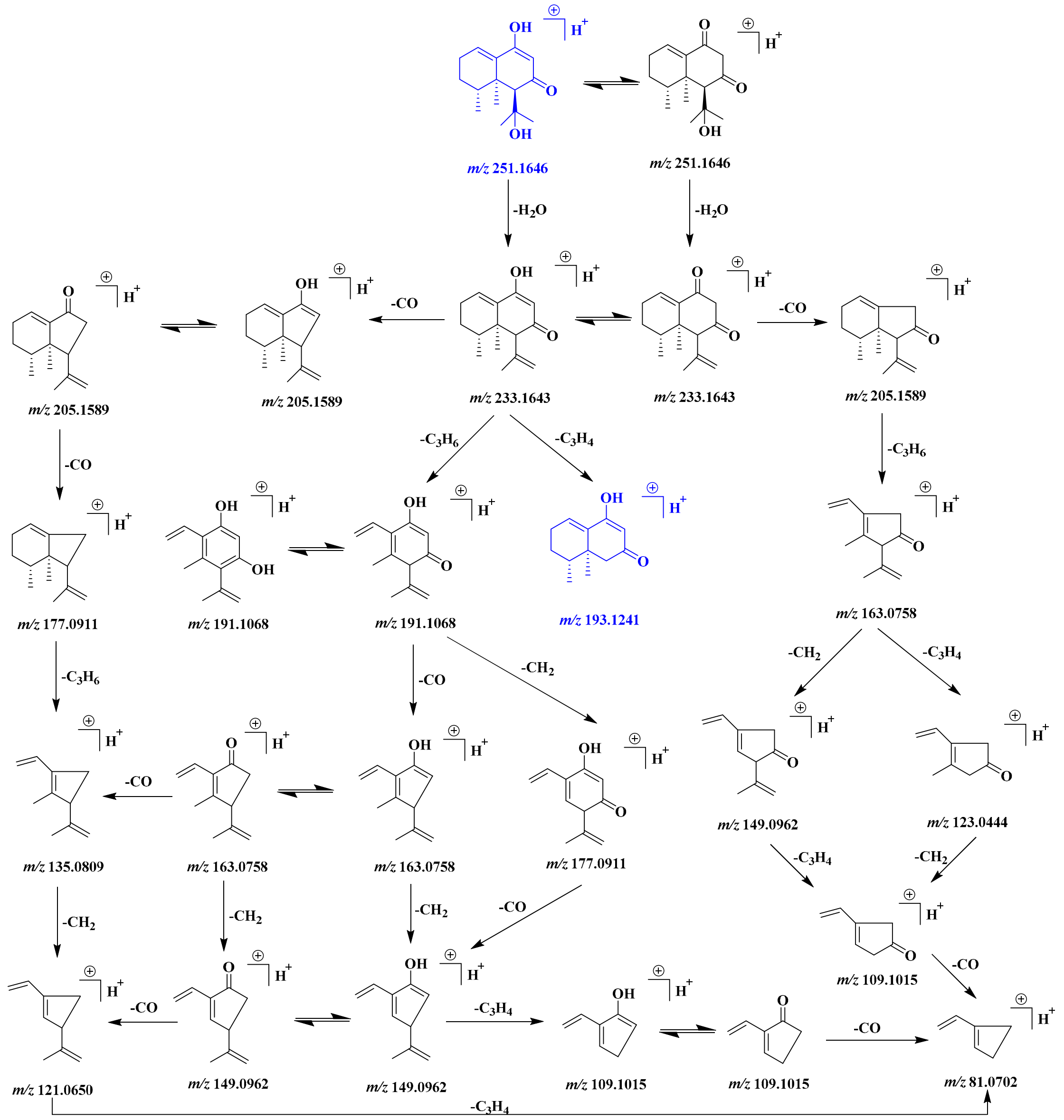

Figure S16 Mass spectrometry fragmentation pathway of 2–deoxokanshone L (positive ion mode)

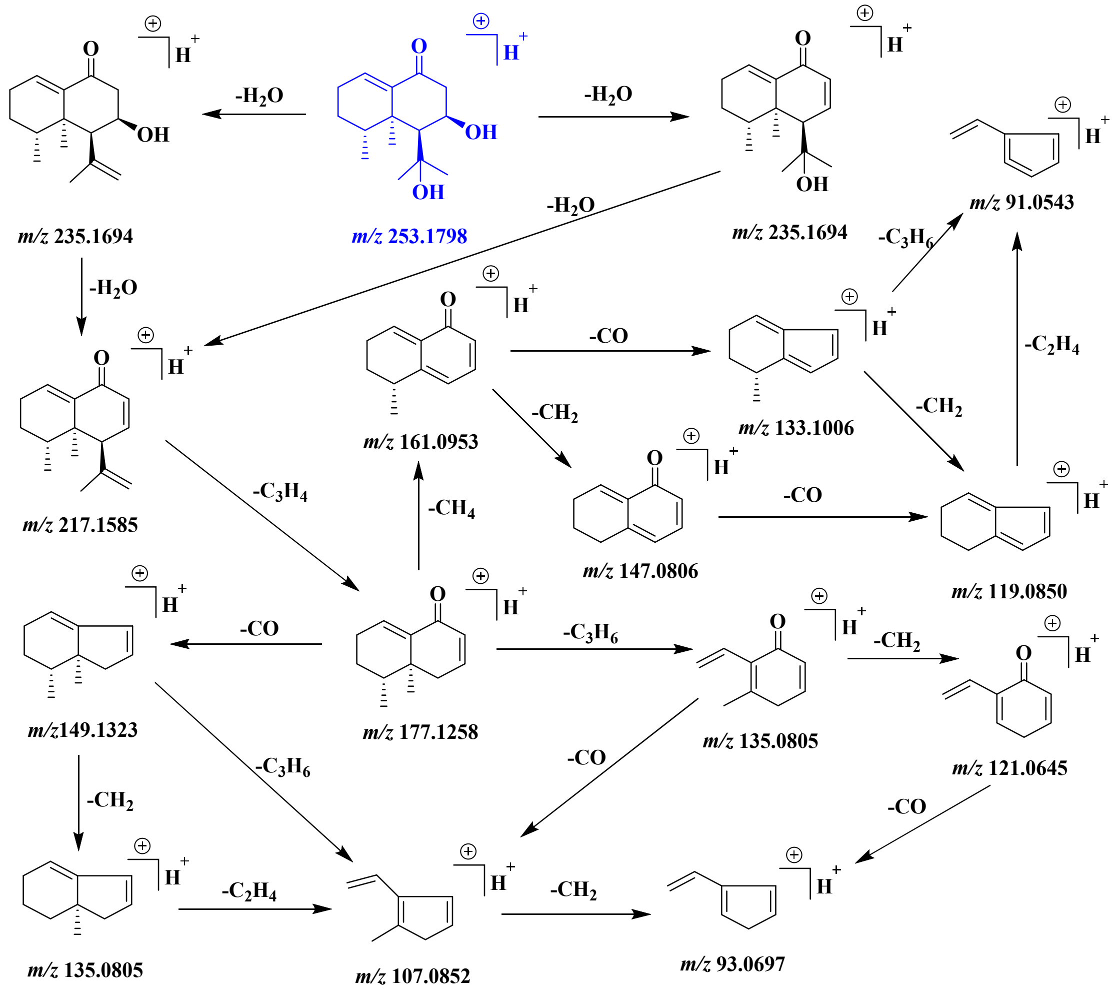

Figure S17 Mass spectrometry fragmentation pathway of nardosinonediol (positive ion mode)

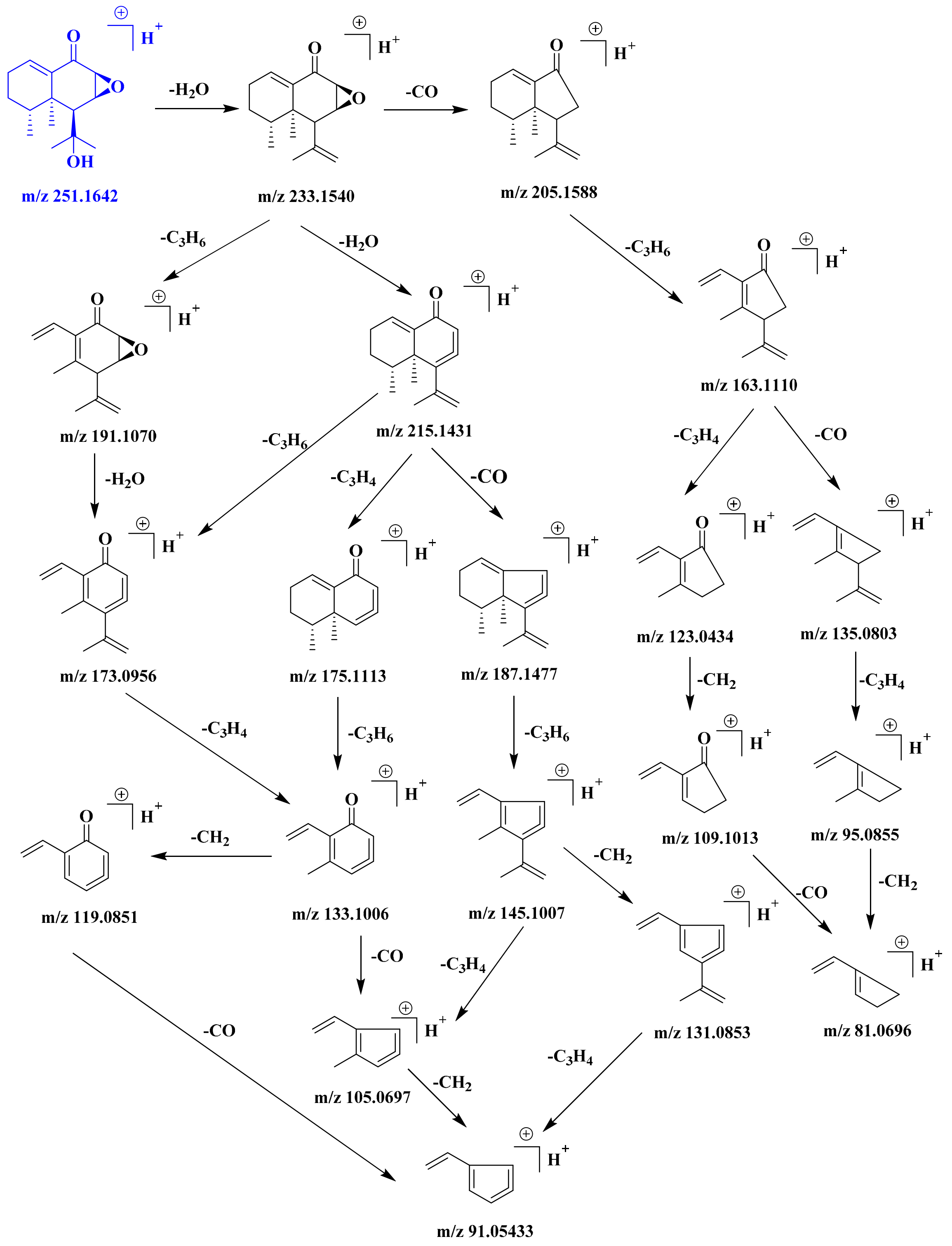

Figure S18 Mass spectrometry fragmentation pathway of isonardosinone (positive ion mode)

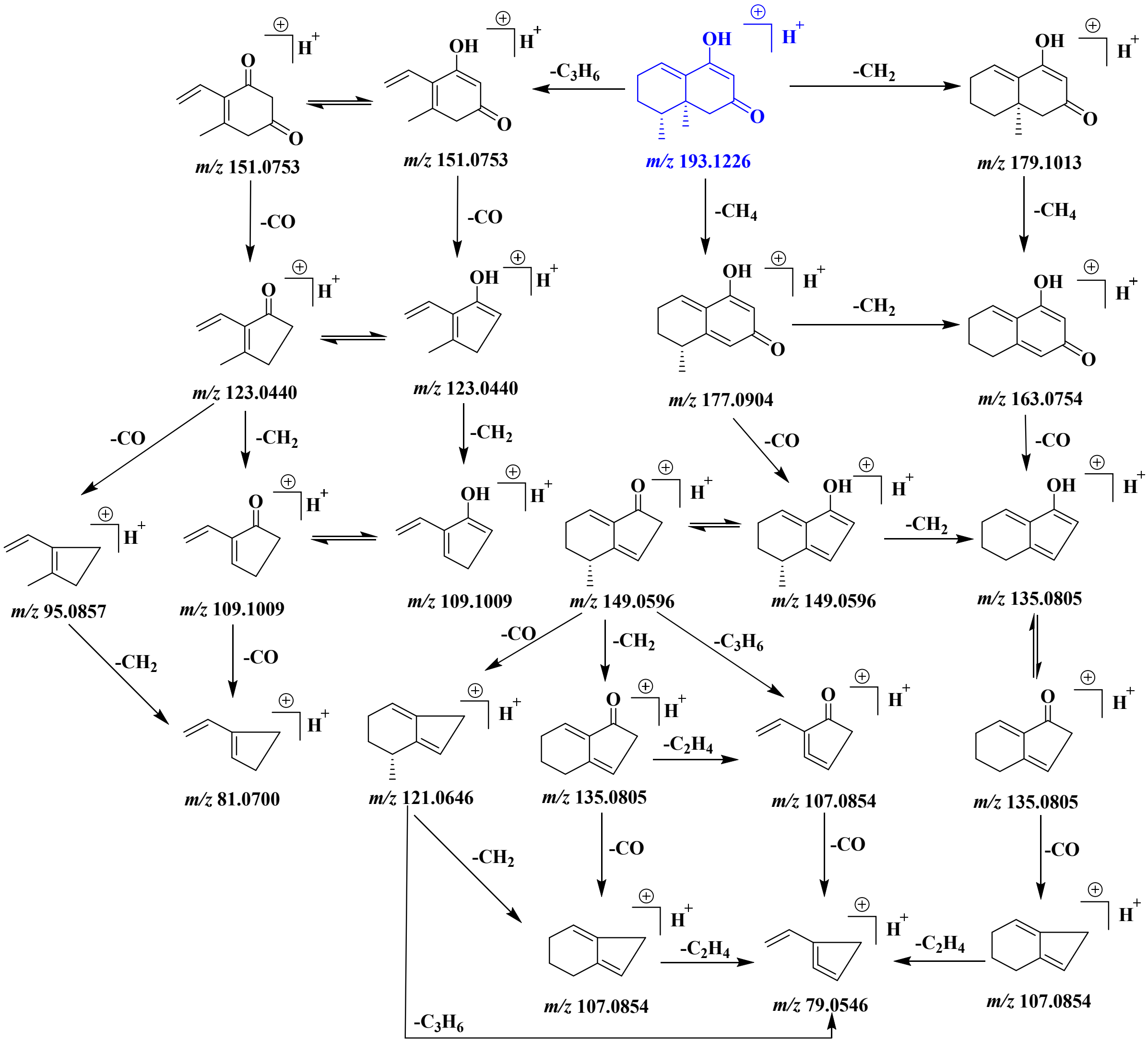

Figure S19 Mass spectrometry fragmentation pathway of 2–deoxokanshone M (positive ion mode)

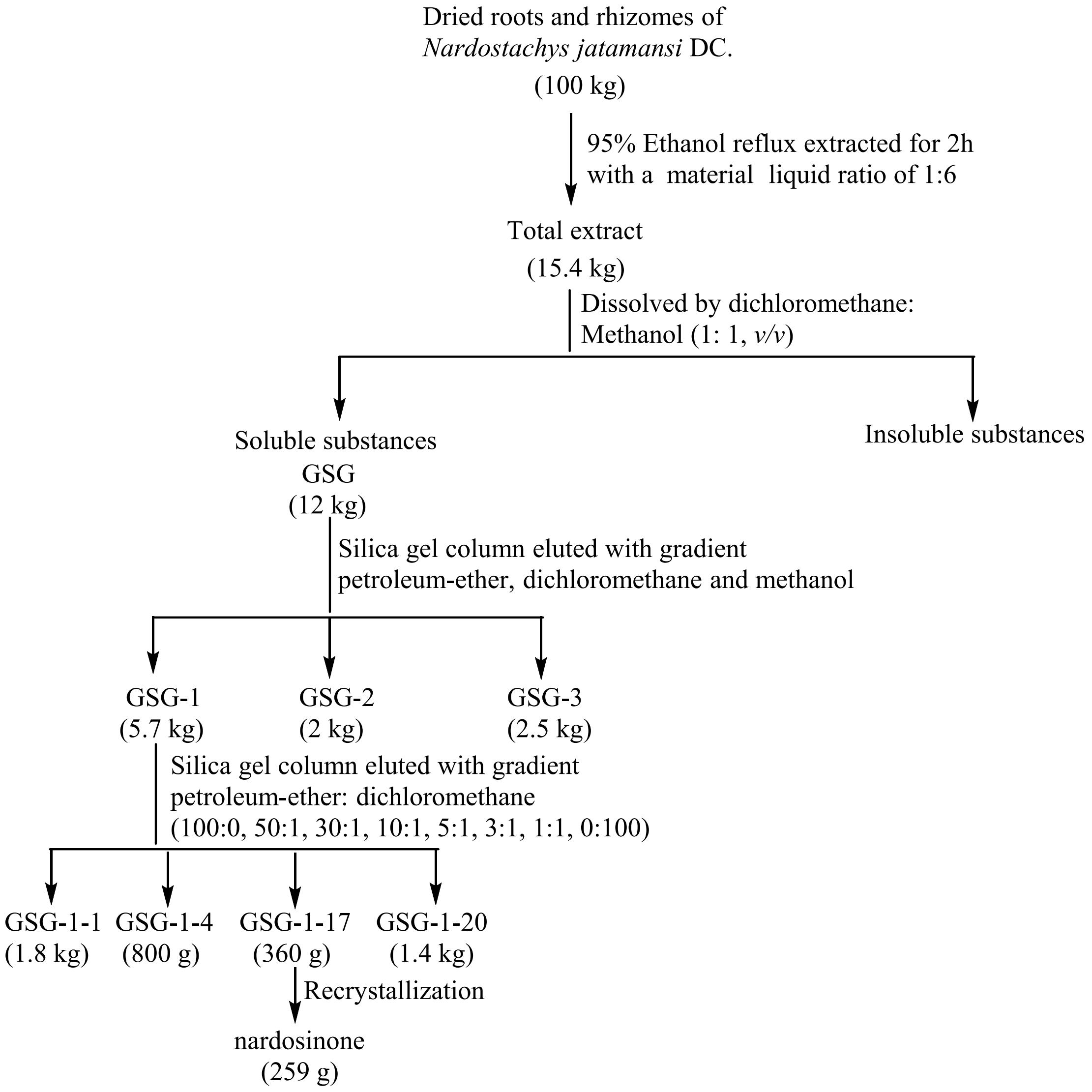

Figure S20 The separation process diagram of nardosinone

Table S1 Characterization of nardosinone and its degradation products in three different conditions by using UHPLC–DAD/ESI–Q–TOF MS

Identification	RT (min)	Formula	Molecular weight	Mass ion (MS)	Fragments	Source
Desoxo-narchinol A (**2**)	8.22	$C_{12}H_{16}O_2$	192.1150	193.1239	175.1117 $[M+H-H_2O]^+$	HT;
				$[M+H]^+$	151.1112 $[M+H-C_3H_6]^+$	SGF;
					147.0797 $[M+H-H_2O-CO]^+$	SGF-A;
					133.1010 $[M+H-H_2O-CO-CH_2]^+$; $[M+H-H_2O-C_3H_6]^+$	SGF-B;
					123.0796 $[M+H-C_3H_6-CO]^+$	SIF
2–Deoxokanshone L (**3**)	11.86	$C_{15}H_{22}O_3$	250.1569	251.1646	233.1643 $[M+H-H_2O]^+$	HT;
				$[M+H]^+$	205.1589 $[M+H-H_2O-CO]^+$	SGF;
					193.1241 $[M+H-H_2O-C_3H_4]^+$	SGF-A;
					191.1068 $[M+H-H_2O-C_3H_6]^+$	SGF-B;
					177.0911 $[M+H-H_2O-CO-CO]^+$; $[M+H-H_2O-C_3H_6-CH_2]^+$	SIF
					163.0758 $[M+H-H_2O-CO-C_3H_6]^+$;	
					149.0962 $[M+H-H_2O-C_3H_6-CO-CH_2]^+$;	
					135.0809 $[M+H-H_2O-CO-CO-C_3H_6]^+$;	
					123.0444 $[M+H-H_2O-CO-C_3H_6-C_3H_4]^+$	
					121.0650 $[M+H-H_2O-CO-CO-C_3H_6-CH_2]^+$;	
					109.1015 $[M+H-H_2O-C_3H_6-CH_2-CO-C_3H_4]^+$;	
Nardosinonediol	14.33	$C_{15}H_{24}O_3$	252.1725	235.1694	217.1585 $[M+H-H_2O-H_2O]^+$	SIF
				$[M+H-H_2O]^+$	177.1258 $[M+H-H_2O-H_2O-C_3H_4]^+$	
					149.0960 $[M+H-H_2O-H_2O-C_3H_4-CO]^+$	

					135.0805 $[M+H-H_2O-H_2O-C_3H_4-C_3H_6]^+$; $[M+H-H_2O-H_2O-C_3H_4-CO-CH_2]^+$	
					121.0645 $[M+H-H_2O-H_2O-C_3H_4-C_3H_6-CH_2]^+$	
					107.0852 $[M+H-H_2O-H_2O-C_3H_4-C_3H_6-CO]^+$; $[M+H-H_2O-H_2O-C_3H_4-CO-CH_2-C_2H_4]^+$	
Isonardosinone (**4**)	17.80	$C_{15}H_{22}O_3$	250.1569	251.1639 $[M+H]^+$	233.1540 $[M+H-H_2O]^+$	HT; SGF-B; SIF
					215.1431 $[M+H-H_2O-H_2O]^+$	
					205.1588 $[M+H-H_2O-CO]^+$	
					187.1477 $[M+H-H_2O-H_2O-CO]^+$	
					175.1113 $[M+H-H_2O-H_2O-C_3H_4]^+$	
					173.0956 $[M+H-H_2O-H_2O-C_3H_6]^+$	
					163.1111 $[M+H-H_2O-CO-C_3H_6]^+$	
					135.0803 $[M+H-H_2O-CO-C_3H_6-CO]^+$	
					133.1006 $[M+H-H_2O-H_2O-C_3H_6-C_3H_4]^+$	
					105.0697 $[M+H-H_2O-H_2O-C_3H_6-C_3H_4-CO]^+$	
2–Deoxokanshone M (**1**)	18.94	$C_{12}H_{16}O_2$	192.1150	193.1226 $[M+H]^+$; 385.2404 $[2M+H]^+$	179.1013 $[M+H-CH_2]^+$	HT; SGF; SGF-A; SGF-B
					175.1122 $[M+H-H_2O]^+$	
					177.0904 $[M+H-CH_4]^+$	
					163.0754 $[M+H-CH_2-CH_4]$+; $[M+H-CH_4-CH_2]^+$	
					151.0753 $[M+H-C_3H_6]^+$	
					149.0596 $[M+H-CH_4-CO]^+$	
					135.0805 $[M+H-CH_2-CH_4-CO]$+; $[M+H-CH_4-CO-CH_2]^+$	
					123.0440 $[M+H-C_3H_6-CO]^+$	

					121.0646 $[M+H-CH_4-CO-CO]^+$	
					109.1009 $[M+H-C_3H_6-CO-CH_2]^+$	
					107.0854 $[M+H-CH_4-CO-CO-CH_2]^+$; $[M+H-CH_4-CO-C_3H_6]^+$	
Nardosinone (**5**)	28.12	$C_{15}H_{22}O_3$	250.1569	251.1646	233.1542 $[M+H-H_2O]^+$	—
				$[M+H]^+$	219.1372 $[M+H-H_2O-CH_2]^+$	
					215.1430 $[M+H-H_2O-H_2O]^+$	
					205.1587 $[M+H-H_2O-CO]^+$	
					193.1226 $[M+H-C_3H_4]^+$	
					191.1429 $[M+H-H_2O-C_3H_6]$+; $[M+H-H_2O-CH_2-CO]^+$	
					177.0905 $[M+H-H_2O-C_3H_6-CH_2]^+$; $[M+H-H_2O-CH_2-CO-CH_2]^+$; $[M+H-H_2O-CO-CO]$+	
					175.1121 $[M+H-C_3H_4-H_2O]^+$	
					163.0750 $[M+H-H_2O-C_3H_6-CO]^+$	
					149.0956 $[M+H-H_2O-CH_2-CO-CH_2-C_2H_4]^+$; $[M+H-H_2O-CH_2-CO-C_3H_6]^+$	
					135.0801 $[M+H-H_2O-CO-CO-CH_2-C_2H_4]^+$	
					123.0440 $[M+H-H_2O-CO-C_3H_6-C_3H_4]^+$	
					109.1010 $[M+H-H_2O-CH_2-CO-CH_2-C_2H_4-C_3H_4]^+$; $[M+H-H_2O-CH_2-CO-C_3H_6-C_3H_4]^+$	

Table S2 The detailed parameters of the NMR measurements

**Name**	**^{1}H-NMR**	**^{13}C-NMR**	**HSQC**	**HMBC**	**^{1}H-^{1}H COSY**
Number of Scans (NS)	16	2838	16	32	16
Receiver Gain (RG)	31.78	55.56	188.54	188.54	188.54
Relaxation Delay (RD/D1)	1.0 s	2.0 s	1.5 s	1.5 s	2.0 s
Spectral Width (SWH)	12019.23 Hz	36231.9 Hz	12019.23 Hz, 36231.9 Hz	12019.23 Hz, 36231.9 Hz	12019.23 Hz, 11990.4 Hz
Acquisition Time (AQ)	2.7263 s	0.9044 s	0.0573 s	0.1704 s	0.0852 s
Spectrometer Frequency (SF)	600.20 MHz	150.95 MHz	600.20 MHz, 150.94 MHz	600.20 MHz, 150.94 MHz	600.21 MHz, 600.21 MHz
Pulse Width (P1)	9.23 μs	10 μs	9.23 μs, 10 μs	9.23 μs, 10 μs	9.23 μs
Line broadening (LB)	0.3 Hz	1 Hz	0 Hz	0 Hz	0 Hz
Nucleus for channel (NUC1)	1 H	13 C	1 H, 13 C	1 H, 13 C	1 H, 1 H
Time domain size (TD)	65536	65536	1024	4096	2048
Number of dummy scans (DS)	0	0	16	16	16
Dwell time (DW)	41.6 μs	13.8 μs	56 μs	41.6 μs	41.6 μs
Pre-scan-delay (DE)	10 μs	18.0 μs	10 μs	10 μs	10 μs
PLW1	25.4480 W	24.194 W	25.4480 W, 24.194 W	25.4480 W, 24.194 W	25.4480 W
SI	65536	65536	1024, 1024	4096, 1024	1024, 1024
FID resolution (FIDRES)	0.367 Hz	1.106 Hz	17.439 Hz	5.859 Hz	11.738 Hz
PC	1	1.4	1.4	1.4	1.4
